# Multi-Dimensional Spectroscopy with Intense Entangled Beams: Entanglement-Enabled Phase Matching in a Collinear Beam Geometry

**DOI:** 10.1021/acs.jpclett.5c02496

**Published:** 2025-11-13

**Authors:** Deependra Jadoun, Upendra Harbola, Vladimir Y. Chernyak, Shaul Mukamel

**Affiliations:** † Division of Chemical Physics and NanoLund, Lund University, 22362 Lund, Sweden; ‡ Department of Chemistry, University of California, Irvine, Irvine, California 92614, United States; § Department of Inorganic and Physical Chemistry, 29120 Indian Institute of Science, Bangalore 560012, India; ∥ Department of Chemistry, 2954 Wayne State University, Detroit, Michigan 48202, United States; ⊥ Department of Mathematics, 2954 Wayne State University, Detroit, Michigan 48202, United States; # Department of Physics and Astronomy, University of California, Irvine, Irvine, California 92614, United States

## Abstract

The experimental realization of quantum molecular spectroscopy with entangled photons remains challenging owing to the low signal-to-noise ratio resulting from the use of low-flux entangled photons. High-flux entangled photons via intense entangled beams can be used to improve the signal-to-noise ratio, but the presence of unentangled photons contaminates the quantum signal stemming from entangled photons. Here, we demonstrate how intense entangled beams can be used in multi-dimensional spectroscopy while retaining the advantage of photon entanglement. Our approach is broadly applicable to odd-ordered nonlinear spectroscopies, and it generates purely quantum spectroscopic signals. The proposed approach allows the recording of desired phase-matched signals even in a collinear beam geometry, which lifts the requirement of complicated beam geometry setups for phase matching in multi-dimensional spectroscopies.

## Graphical abstract

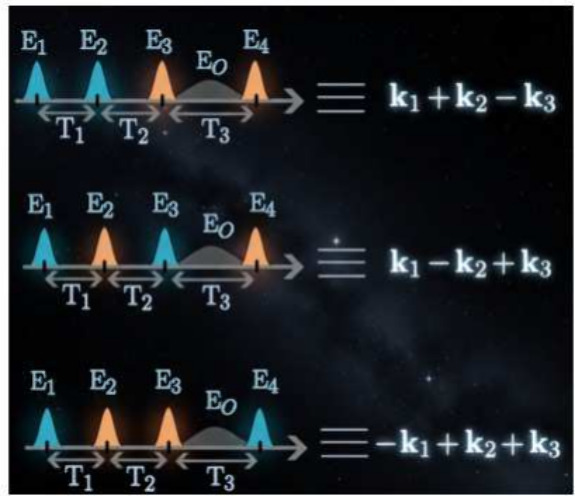

Entangled photons^1^ have emerged as a promising tool in molecular spectroscopy^2^ over the past decades, and multiple theoretical studies concerning pump–probe^3–6^ and multi-dimensional^7,8^ spectroscopies were enabled. An enhanced yield of two-photon absorption with entangled photons has been predicted in molecules.^6,9–13^ Theoretical studies have predicted that entangled photons provide superior spectral and temporal resolutions beyond the classical Fourier limit in pump–probe spectroscopies.^14–16^ Entangled-photon pairs have also been used in multi-dimensional spectroscopy for selectively probing light–matter interaction pathways.^17–21^ However, the experimental utilization of entangled photons in molecular spectroscopies has been challenging since the optimal use of entanglement in spectroscopy requires using low-flux entangled photons, which results in a low signal-to-noise ratio.^22^ Recording such weak signals thus requires the development of highly sensitive detection techniques. Despite significant recent advancements in that direction,^23,24^ spectroscopy with low-flux entangled photons remains challenging.

An obvious approach to improve the signal-to-noise ratio is to use intense entangled beams generated via high-gain parametric-down conversion (PDC), where a large number of pairs of entangled photons interact with the sample under study. This, however, allows the sample to interact with both entangled and unentangled photons, and the spectroscopic signal stemming from entangled photons can be obscured by the signal from unentangled photons. Thus, the utilization of entanglement in spectroscopy requires the development of a protocol that eliminates the classical components of the signal originating from the interaction of the sample with unentangled photons.

In this paper, we propose a method that allows recording purely quantum spectra when intense entangled beams are used in multi-dimensional spectroscopy. The proposed approach can be used in any spectroscopic scheme that uses an even number of light pulses. In multipulse spectroscopic techniques, in general, desired signals are collected in different phase-matching directions. This is not possible in a collinear or nearly collinear setup with classical light. Here, we demonstrate another advantage of using quantum light, which allows us to pick out any desired signal even in a collinear pulse geometry by merely manipulating the sequence of various entangled pulses. We consider a collinear setup for four-wave mixing (FWM), such as 2D electronic spectroscopy (2DES), consisting of non-rephasing (NRP), rephasing (RP), and double quantum-coherence (DQC) signals that can be selectively recorded.

The easiest way to understand the emergence of the classical field component in intense entangled beams is to look at the frequency-dispersed coincidence detection spectrum of entangled beams

1 $$C\left(\omega ,\omega^{\prime }\right)={\left\langle {Ê}_{s}^{\left(-\right)}\left(\omega \right){Ê}_{s}^{\left(+\right)}\left(\omega \right){Ê}_{i}^{\left(-\right)}\left(\omega^{\prime }\right){Ê}_{i}^{\left(+\right)}\left(\omega^{\prime }\right)\right\rangle }_{F}$$

where ${Ê}^{\left(+\right)}\left(t,r\right)$ = ${\left[{Ê}^{\left(-\right)}\left(t,r\right)\right]}^{†}$ = $ê\int d\omega {Ê}^{\left(+\right)}\left(\omega \right)e^{-i\omega t}e^{ik\cdot r}$ , with ê being the polarization vector, **k** being the field wave vector, **r** being the position vector, and the subscript “s” (“i”) represents the signal (idler) beam. ${Ê}^{\left(+\right)}$ ( ${Ê}^{\left(-\right)}$ ) is the positive (negative) frequency component of the field operator ( Ê ) such that

2 $$Ê\left(t,r\right)={Ê}^{\left(+\right)}\left(t,r\right)+{Ê}^{\left(-\right)}\left(t,r\right)$$

Note that our definition of the electric field operator implies that the positive frequency component corresponds to photon annihilation and vice versa. The expectation value in eq 1 is over the entangled field state, as represented by the subscript “F”.

Since the entangled field generated by high-gain PDC has Gaussian statistics,^25^ one can use Wick’s theorem to decompose a multi-point field correlation function into a product of two-point correlation functions in terms of the intensity–intensity correlation,

3 $$C\left(\omega ,\omega^{\prime }\right)=\underset{\mathrm{intra‐mode correlations}}{\underset{︸}{{\left\langle {Ê}_{s}^{\left(-\right)}\left(\omega \right){Ê}_{s}^{\left(+\right)}\left(\omega \right)\right\rangle }_{F}{\left\langle {Ê}_{i}^{\left(-\right)}\left(\omega^{\prime }\right){Ê}_{i}^{\left(+\right)}\left(\omega^{\prime }\right)\right\rangle }_{F}\;}}+\underset{\mathrm{inter‐mode correlations}}{\underset{︸}{{\left\langle {Ê}_{s}^{\left(-\right)}\left(\omega \right){Ê}_{i}^{\left(-\right)}\left(\omega^{\prime }\right)\right\rangle }_{F}{\left\langle {Ê}_{s}^{\left(+\right)}\left(\omega \right){Ê}_{i}^{\left(+\right)}\left(\omega^{\prime }\right)\right\rangle }_{F}\;}}$$

where each correlation function in the first term to the right-hand side of the above equation consists of electric field operators for the same beam (either “s” or “i”) and is thus called an intra-mode correlation, whereas each correlation function in the second term consists of electric field operators for different beams and is thus denoted as an inter-mode correlation. Contributions such as $\left\langle {Ê}_{s}^{\left(+\right)}\left(\omega \right){Ê}_{i}^{\left(-\right)}\left(\omega^{\prime }\right)\right\rangle$ are not present in eq 3 since they vanish (see ref 13). The inter-mode term ( $\left\langle {Ê}_{s/i}^{\left(\pm \right)}{Ê}_{i/s}^{\left(\pm \right)}\right\rangle$ ) carries the information regarding quantum entanglement, whereas the intra-mode term ( $\left\langle {Ê}_{s/i}^{\left(\pm \right)}{Ê}_{s/i}^{\left(\mp \right)}\right\rangle$ ) represents classical correlations. Frequency-dispersed inter- and intra-mode terms are shown in Figure 1 for low-, mid-, and high-gain PDC. The PDC process is treated non-perturbatively to compute the correlation functions in eq 3, and the relevant details regarding the numerical calculations can be found in ref 13. As shown in the upper panel of Figure 1, the inter-mode term shows frequency entanglement (anti-correlation) between the involved modes for each gain. The frequency anti-correlation between the entangled modes is tight for low gain, as can be seen by the narrow width along the diagonal in Figure 1a. For mid- and high gains, the width along the diagonal increases due to the increase in the pump intensity, which results in a comparatively weaker entanglement. Even though the field strength is multiple orders higher than the low- and mid-gain cases, a strong frequency anti-correlation is present in the inter-mode term for the high-gain case, as shown in Figure 1c. Thus, entangled beams generated by high-gain PDC can provide a much better signal-to-noise ratio than low-flux entangled photons without a significant loss of entanglement.

**Figure 1:**
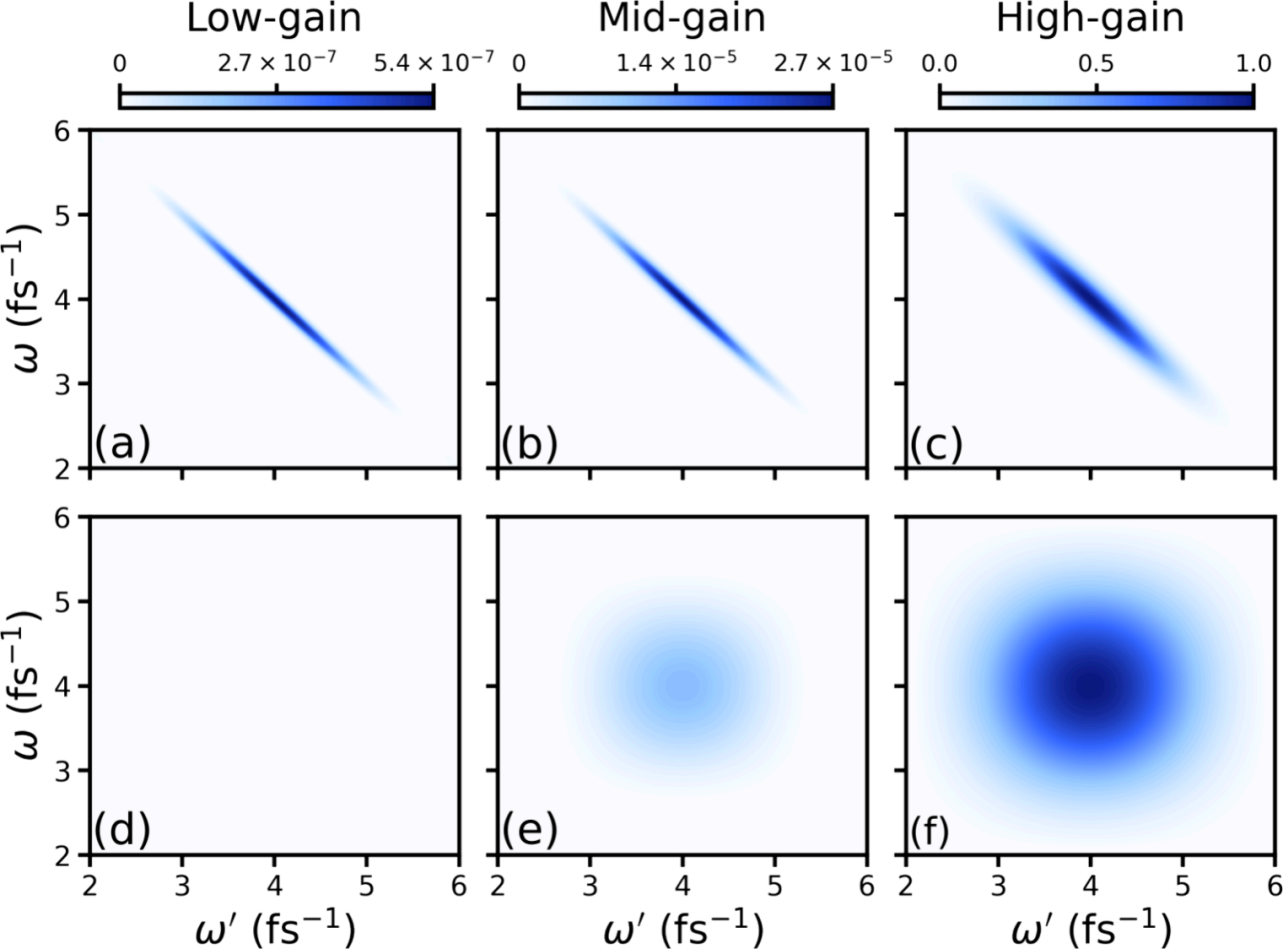
Frequency-dispersed coincidence-detection plots for different gains from PDC computed using eq 3 are shown. Inter- and intra-mode correlations are plotted in the upper and lower panels, respectively, for the low-, mid-, and high-gain PDC. Intensities of all the field components for the three different gains are normalized with respect to the quantum component of the high-gain process.

The intra-mode contribution does not show any correlation between the modes, and it is negligible compared to the inter-mode contribution for low gain (Figure 1d). With an increase in the pump intensity, however, the two field contributions (inter- and intra-mode) become comparable at high gain, as shown in Figure 1c and f. The field profile of the intra-mode contribution is shown in Figure 1f for high-gain PDC, and it resembles that of a coherent state (Gaussian) of light.^26^ Thus, the intra-mode term contributes to the emergence of the classical signal component when intense entangled beams are used and tends to diminish the quantum contribution, which also results in a reduced entanglement transfer to the matter.^27^ Covariance plots similar to Figure 1 have been obtained experimentally.^28–30^ We have recently shown that the intra-mode field component can be eliminated in two-dimensional electronic spectroscopy^31^ with degenerate entangled beams generated via a single PDC by manipulating the pulse sequence. Here, we propose a generic approach that can be used to obtain purely quantum signals in multi-dimensional *n*-wave mixing spectroscopies with both degenerate and non-degenerate frequency-entangled beams using multiple PDC sources.

The total Hamiltonian reads

4 $$Ĥ={Ĥ}_{M}+{Ĥ}_{F}+{Ĥ}_{\mathrm{int}},,{Ĥ}_{\mathrm{int}}=-\mathrm{\mu ̂}\cdot Ê$$

where ${Ĥ}_{M}$ is the molecular Hamiltonian, ${Ĥ}_{F}$ is the field Hamiltonian, and μ̂ represents the operator for molecular excitations and de-excitations via the molecule–field interaction, which is embedded in ${Ĥ}_{\mathrm{int}}$ within the dipole approximation.

In a *n*-wave mixing process, *n* – 1 light pulses interact with the sample to create a nonlinear response, which results in the emission of the *n*th output signal field. In general, the output signal field is weak and heterodyne-detected, where the signal field interferes with a local-oscillator field prior to detection. The signal can be defined as the first-order coherence of the heterodyned output signal field and, in the Heisenberg picture, reads as^32^

5 $$S\left(\tau \right)=\int dt\left[{\left\langle {Ê}_{D,R}^{\left(-\right)}\left(t+\tau ,r_{D}\right){Ê}_{D,L}^{\left(+\right)}\left(t,r_{D}\right)\right\rangle }_{T}+{\left\langle {Ê}_{D,L}^{\left(+\right)}\left(t+\tau ,r_{D}\right){Ê}_{D,R}^{\left(-\right)}\left(t,r_{D}\right)\right\rangle }_{T}\right]$$

where ${Ê}_{D}={Ê}_{n}+{Ê}_{O}$ , with ${Ê}_{O}$ being the output field resulting from the *n*-wave mixing that is heterodyned using the local oscillator field ${Ê}_{n}$ , ${Ê}_{D}$ is the total field at the detector position **r**_D_, ${\left\langle ...\right\rangle }_{T}$ represents the expectation value over the total Hamiltonian ( Ĥ ) and is a shorthand representation of Tr­{...ρ_0_}, with ρ_0_ = $\rho_{0}^{M}\rho_{0}^{F}$ representing the total density matrix at *t* = 0, where $\rho_{0}^{M}$ ( $\rho_{0}^{F}$ ) is the initial matter (field) density matrix. One can Fourier transform the signal expression over delay τ to obtain a frequency dispersed signal. Note that the electric field operators in the above expression are Liouville space super-operators,^100^ and the subscripts R and L denote the right and left operation of a super-operator on the system density matrix, respectively.

Converting eq 5 from the Heisenberg picture to the interaction picture and performing the perturbative expansion in the interaction Hamiltonian leads to the following expression for the *n*-wave mixing signal^33^

6 $$S\left(\tau \right)={\left(-\frac{i}{\hbar }\right)}^{n}\int dtdt_{n}...dt_{1}\langle \left({Ê}_{n,R}^{\left(-\right)}\left(t+\tau ,r_{D}\right){Ê}_{O,L}^{\left(+\right)}\left(t,r_{D}\right)+{Ê}_{O,L}^{\left(+\right)}\left(t+\tau ,r_{D}\right){Ê}_{n,R}^{\left(-\right)}\left(t,r_{D}\right)\right)\times {{Ĥ}_{-,\mathrm{int}}\left(t_{n}\right)...{Ĥ}_{-,\mathrm{int}}\left(t_{2}\right){Ĥ}_{-,\mathrm{int}}\left(t_{1}\right)\rangle }_{M+F}$$

where ${\left\langle {Ĥ}_{-,\mathrm{int}}\left(t_{1}\right)\right\rangle }_{M+F}=\mathrm{Tr}\left\{\left({Ĥ}_{L,\mathrm{int}}\left(t_{1}\right)-{Ĥ}_{R,\mathrm{int}}\left(t_{1}\right)\right)\rho_{0}\right\}$ and the subscript M + F represents the expectation value over the matter and field degrees of freedom. Note that the time evolution of operators is in the interaction picture with respect to the Hamiltonian ${Ĥ}_{\mathrm{int}}$ .

Substituting the electric field operators from eq 2 in eq 6 gives rise to several components of an *n*-wave mixing signal. Contributions to the *n*-wave mixing process can be classified based on positive and negative frequency components, which represent the signal expression for a specific field interaction. These relate to different phase-matched components satisfying conservation of momentum: **k**_*O*_ = ±**k**_*n*–1_ ± ... ± **k**_2_ ± **k**_1_. The collinear propagation of the incoming beams results in a *n*-wave mixing signal composed of signals from all of the phase-matching conditions. Recording the *n*-wave mixing signal corresponding to a specific phase-matching contribution requires control over the propagation direction of the incoming pulses. Each phase-matched component consists of contributions from different Liouville space pathways that differ in the evolution of the matter density matrix due to time-ordered interactions of the electric field operators (see ref 33).

We shall use FWM to demonstrate the proposed scheme, which can be extended to more complicated spectroscopies in a straightforward manner. For a FWM process, eq 6 takes the following form:

$$S_{\mathrm{FWM}}\left(\tau \right)={\left(-\frac{i}{\hbar }\right)}^{4}\int dtdt_{4}...dt_{1}\langle \left({Ê}_{4,R}^{\left(-\right)}\left(t+\tau ,r_{D}\right){Ê}_{O,L}^{\left(+\right)}\left(t,r_{D}\right)+{Ê}_{O,L}^{\left(+\right)}\left(t+\tau ,r_{D}\right){Ê}_{4,R}^{\left(-\right)}\left(t,r_{D}\right)\right)\times {{Ĥ}_{-,\mathrm{int}}\left(t_{4}\right){Ĥ}_{-,\mathrm{int}}\left(t_{3}\right){Ĥ}_{-,\mathrm{int}}\left(t_{2}\right){Ĥ}_{-,\mathrm{int}}\left(t_{1}\right)\rangle }_{M+F}$$ 7

Figure 2 shows the pulse sequence along with the detection scheme for the FWM process under consideration, where three pulses interact with the sample/molecule (“M”), which results in the emission of the output signal field **E**_O_ that is heterodyned with the local-oscillator field **E**_4_ prior to detection. Substituting for the interaction Hamiltonian ${Ĥ}_{\mathrm{int}}=\mathrm{\mu ̂}\cdot Ê$ , the FWM signal can be recast as

$$S_{\mathrm{FWM}}\left(\tau \right)=\frac{{\left(-1\right)}^{m}}{\hbar^{4}}\int dtdt_{4}...dt_{1}{\left\langle {\mathrm{\mu ̂}}_{L}\left(t_{4}\right){\mathrm{\mu ̂}}_{R,L}\left(t_{3}\right){\mathrm{\mu ̂}}_{R/L}\left(t_{2}\right){\mathrm{\mu ̂}}_{R/L}\left(t_{1}\right)\right\rangle }_{M}\langle ({Ê}_{4,R}^{\left(-\right)}\left(t+\tau ,r_{D}\right){Ê}_{O,L}^{\left(+\right)}\left(t,r_{D}\right)+{Ê}_{O,L}^{\left(+\right)}\left(t+\tau ,r_{D}\right){Ê}_{4,R}^{\left(-\right)}\left(t,r_{D}\right)){Ê}_{O,L}^{\left(-\right)}\left(t_{4},r_{M}\right){Ê}_{3,R/L}\left(t_{3},r_{M}\right){{Ê}_{2,R/L}\left(t_{2},r_{M}\right){Ê}_{1,R/L}\left(t_{1},r_{M}\right)\rangle }_{F}$$ 8

where *m* is the number of *L* super-operators in the matter correlation function, μ̂ is the transition dipole-moment operator, and **r**_M_ is the position vector corresponding to the sample. The six-point field correlation function in the above expression contains both inter- and intra-mode field contributions that are responsible for the quantum and classical components of the signal, respectively.

**Figure 2:**
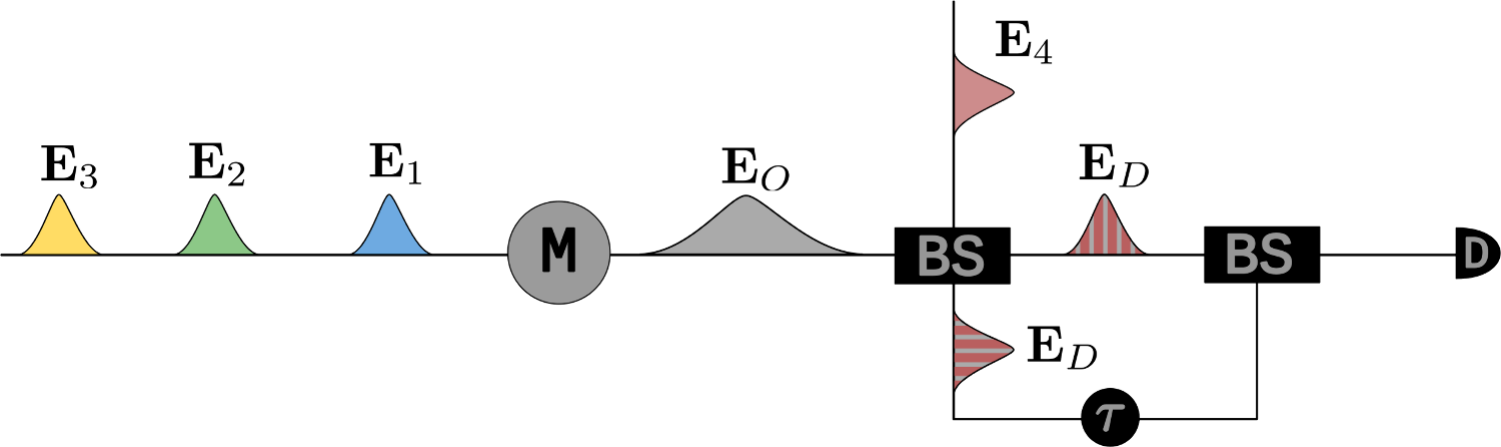
Pulse scheme depicting the first-order coherence detection in a collinear FWM heterodyne signal is shown. “M” represents the molecule/sample under study, and “BS” represents the beam splitter used for mixing the output signal field with the heterodyne beam as well as the first-order coherence pulses.

Using Wick’s theorem, it can be recast in terms of two-point field correlations

$$\langle {Ê}_{O,L}^{\left(+\right)}\left(t+\tau ,r_{D}\right){{Ê}_{4,R}^{\left(-\right)}\left(t,r_{D}\right){Ê}_{O,L}^{\left(-\right)}\left(t_{4},r_{M}\right){Ê}_{3,R/L}\left(t_{3},r_{M}\right){Ê}_{2,R/L}\left(t_{2},r_{M}\right){Ê}_{1,R/L}\left(t_{1},r_{M}\right)\rangle }_{F}={\left\langle {Ê}_{O,L}^{\left(+\right)}\left(t+\tau ,r_{D}\right){Ê}_{O,L}^{\left(-\right)}\left(t_{4},r_{M}\right)\right\rangle }_{0}\times [{\left\langle {Ê}_{4,R}^{\left(-\right)}\left(t,r_{D}\right){Ê}_{3,R/L}\left(t_{3},r_{M}\right)\right\rangle }_{F}{\left\langle {Ê}_{2,R/L}\left(t_{2},r_{M}\right){Ê}_{1,R/L}\left(t_{1},r_{M}\right)\right\rangle }_{F}+{\left\langle {Ê}_{4,R}^{\left(-\right)}\left(t,r_{D}\right){Ê}_{2,R/L}\left(t_{2},r_{M}\right)\right\rangle }_{F}{\left\langle {Ê}_{3,R/L}\left(t_{3},r_{M}\right){Ê}_{1,R/L}\left(t_{1},r_{M}\right)\right\rangle }_{F}+{\left\langle {Ê}_{4,R}^{\left(-\right)}\left(t,r_{D}\right){Ê}_{1,R/L}\left(t_{1},r_{M}\right)\right\rangle }_{F}{\left\langle {Ê}_{3,R/L}\left(t_{3},r_{M}\right){Ê}_{2,R/L}\left(t_{2},r_{M}\right)\right\rangle }_{F}]$$ 9

where the two-point correlation function of the output field ${Ê}_{O}$ is evaluated in the vacuum state indicated by ${\left\langle ...\right\rangle }_{0}$ , and the corresponding correlation function leads to a Dirac delta.

In eq 9, each field operator is a sum of “s” and “i” modes, and the survival of each term inside the square bracket depends on the frequency component (positive or negative) and the field mode (“s” or “i”) of the involved electric field operators. Terms with different field modes (inter-mode terms) survive for the same frequency components of the involved electric field operators and contribute to the quantum signal component, whereas terms with the same field modes (intra-mode terms) survive for the opposite frequency components (positive and negative) of the involved electric field operators and contribute to the classical signal component. However, one can eliminate the intra-mode component entirely if entangled beams generated by two PDC processes are used in FWM such that no two electric field operators belong to the same field mode, as discussed below.

When entangled beams generated from one PDC process are used for the first two light pulses such that **E**_1_ = **E**_s1_ and **E**_2_ = **E**_i1_ and the entangled beams generated from the other PDC process are used for the last two light pulses such that **E**_3_ = **E**_s2_ and **E**_4_ = **E**_i2_, only the first term in eq 9 is non-zero since $\left\langle {Ê}_{2}{Ê}_{1}\right\rangle$ = $\left\langle {Ê}_{i1}{Ê}_{s1}\right\rangle$ and $\left\langle {Ê}_{4}{Ê}_{3}\right\rangle$ = $\left\langle {Ê}_{i2}{Ê}_{s2}\right\rangle$ . Correlations in the remaining two terms, such as $\left\langle {Ê}_{4}{Ê}_{2}\right\rangle$ = $\left\langle {Ê}_{i2}{Ê}_{i1}\right\rangle$ , vanish since the involved field modes are not correlated. The corresponding pulse sequence is depicted in Figure 3a, where the same-colored pulses are entangled and are generated independently from the different-colored pulses. Moreover, since the signal expression has the negative frequency component of ${Ê}_{4}$ , the correlation function $\left\langle {Ê}_{4}^{\left(-\right)}{Ê}_{3}\right\rangle$ = $\left\langle {Ê}_{i2}^{\left(-\right)}{Ê}_{s2}\right\rangle$ is non-zero only for the negative frequency component of ${Ê}_{3}$ . Consequently, the phase-matching condition (±**k**_1_ ± **k**_2_ – **k**_3_ – **k**_O_ = 0) is only satisfied when the signal expression contains positive-frequency components of the electric field operators for the first two light pulses ( ${Ê}_{1}^{\left(+\right)}$ and ${Ê}_{2}^{\left(+\right)}$ ). This leads to phase matching, **k**_O_ = **k**_1_ + **k**_2_ – **k**_3_, that belongs to the DQC pathway, while other pathways (NRP and RP) are removed. A Liouville pathway corresponding to DQC is plotted in Figure 3b, where black (gray) double strands represent the interaction of light with the sample (detector). Arrows representing electric field operators are connected to denote a two-point correlation function in eq 9, where a filled circle represents a PDC process.

**Figure 3:**
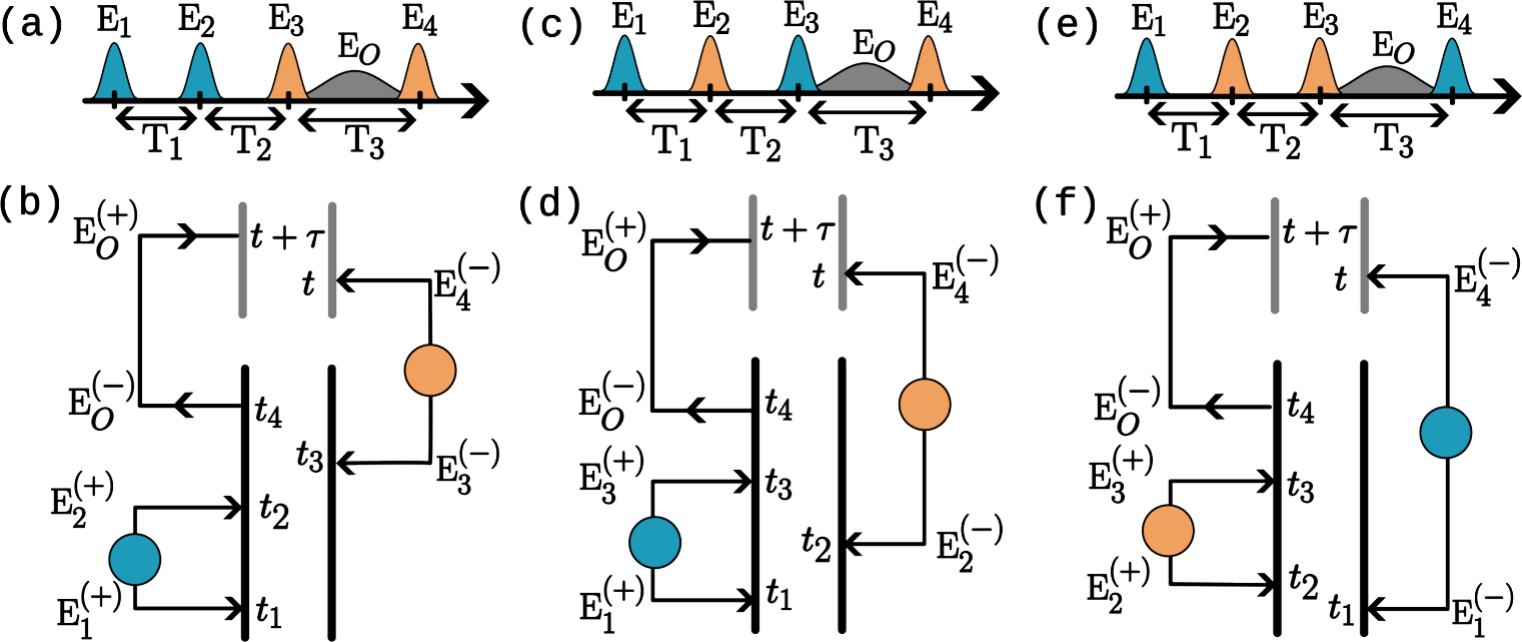
Collinear pulse sequences for detecting (a) DQC (**k**_1_ + **k**_2_ – **k**_3_), (c) NRP (**k**_1_ – **k**_2_ + **k**_3_), and (e) RP (−**k**_1_ + **k**_2_ + **k**_3_) phase-matching signals are shown. Pulses with the same color are entangled in the depicted pulse sequences, and different-colored pulses are generated independently. Ladder diagrams corresponding to one of the pathways for DQC, NRP, and RP phase-matched signals are shown in panels b, d, and f, respectively. Two-point field correlators are represented by connected arrows in the ladder diagrams, where a filled circle represents that the fields are generated by a PDC process and a missing circle indicates that the field is in a vacuum state prior to the light–matter interaction.

When entangled beams from one PDC process are used for the first and third light pulses (**E**_1_ = **E**_s1_ and **E**_3_ = **E**_i1_) and entangled beams from the other PDC process are used for the second and fourth pulses (**E**_2_ = **E**_s2_ and **E**_4_ = **E**_i2_), only the second term in eq 9 survives. This gives rise to the signal corresponding to the NRP pathway since the total field correlation function is non-zero only for the phase-matching condition **k**_O_ = **k**_1_ – **k**_2_ + **k**_3_. The corresponding pulse sequence is shown in Figure 3c. The excited-state absorption (ESA) Liouville light–matter interaction pathway for NRP phase-matching is shown in Figure 3d. Similarly, the third term in eq 9 survives only when the second and third pulses are entangled and the first and the fourth pulses are entangled. This gives rise to the signal corresponding to the RP phase matching (**k**_O_ = −**k**_1_ + **k**_2_ + **k**_3_). The corresponding pulse ordering and ladder diagrams are shown in Figure 3e and f, respectively.

Thus, the entangled field can be used to control the phase-matched signal generated in a FWM signal, and the desired signal (NRP, RP, or DQC) can be recorded in a collinear beam geometry, just by changing the order/sequence of involved entangled pulses. For a collinear beam geometry with classical light pulses, the output signal is a convolution of signals corresponding to each phase-matching condition. Additional steps, such as phase cycling,^34,35^ are necessary in order to suppress the spectroscopic signals from unwanted pathways when pulses in a collinear beam geometry are used. Alternatively, one can use noncollinear beam geometries to selectively record each phase-matched signal.^36–38^ However, this requires precise control over the geometry of the incoming pulses as well as the position of the detector, which makes such setups highly sensitive to external influences. Achieving phase matching with intense entangled beams is much simpler since the collinear beam propagation is sufficient and the order or sequence of entangled beams can be used to record a desired signal. However, the delay control of entangled beams is more complicated than classical pulses, since it depends on the length of the PDC crystal. Since the generation of entangled photons inside a PDC crystal is spontaneous, the delay at which the two entangled photons/beams exit a PDC crystal is statistical. This can be studied using Glauber’s second-order coherence function *g* ^(2)^ as a function of delay in the coincidence detection.^13^

Estimating the strength of a 2DES signal generated with entangled beams is crucial for its practical implementation. The strength of a 2DES signal depends on the field correlation function and the matter response for both classical and quantum spectroscopies. Thus, the strength of a 2DES signal is identical for both spectroscopies as long as the amplitudes of the electric fields in the field correlation function are the same for both classical and quantum spectroscopies. However, the field amplitude of classical (coherent) pulses can be directly controlled, which is not the case with entangled beams since it depends on the length of the PDC crystal and the amplitude of the pump pulse used to drive the PDC process. The amplitude of an entangled field is ${Ê}_{s/i}\propto \chi^{\left(2\right)}{Ê}_{P}$ , where ${Ê}_{P}$ is the pump–pulse amplitude and χ^(2)^ is the second-order nonlinearity constant for the PDC crystal. In order to obtain a quantum spectroscopic signal that is as strong as a classical signal, one can tune the intensity of the pump pulse for a given PDC crystal to match the amplitude of the entangled field to that of a classical coherent pulse.

In conclusion, we have presented a framework that allows using intense entangled beams to generate purely quantum spectra in an odd-ordered multi-dimensional spectroscopy with a much better signal-to-noise ratio compared to when low-flux etangled photons are used. Entangled beams generated by independent PDC processes allow the elimination of the classical contribution. It is worth noting that the proposed method can be realized using a single nonlinear crystal instead of multiple as long as pairs of entangled beams are generated in distinct PDC events. This can be achieved by using a set of temporally well-separated pulses in the PDC process, such that each pulse generates an independent pair of intense entangled beams. The sequence of entangled beams can be tailored for selectively recording signals from the desired phase-matching pathways, even in a collinear beam geometry. This study paves the way for the development of integrated and waveguide-compatible spectroscopic setups and lifts the requirement of using complicated noncollinear beam geometries for recording phase-matched signals.

## Data Availability

The data underlying the results presented in this paper can be obtained from the authors upon reasonable request.
